# Why does transcultural consultation matter?

**DOI:** 10.1192/j.eurpsy.2021.1812

**Published:** 2021-08-13

**Authors:** A. Quintão, C. Santos, M.D. Urzal, I. Donas-Boto, F. Azevedo, M.M. Lemos, F. Coelho, J. Vian

**Affiliations:** 1 Psychiatry Department, Ocidental Lisbon Hospital Center, Lisboa, Portugal; 2 Psychiatry Department, North Lisbon Hospital Center, Lisboa, Portugal

**Keywords:** transcultural, cultural consultation

## Abstract

**Introduction:**

Psychiatrists should be aware of the new challenges and needs that globalization poses.

**Objectives:**

To highlight the need for a culturally sensitive approach to mental health.

**Methods:**

Non-systematic review of “cultural consultation” on PubMed.

**Results:**

Most people assume a direct connection between pathophysiology and clinical symptoms. However, evidence shows that the translation of pathophysiology and psychopathology into specific symptoms is mediated by cognitive processes and social interactions, which reflect models/practices specific to our culture. Patients focus on specific aspects of being sick, reinforced by cultural narratives or to fit expectations. Thus, people from different cultural backgrounds might have trouble communicating; cultural idioms of distress can be misinterpreted. The role of structural violence bestowed upon cultural minorities, which leads to discrimination and social exclusion, has extensively been studied as a risk factor for mental illness. Furthermore, ignoring cultural differences and diversity has been shown to contribute to healthcare disparities, hampering access to care and diminishing the quality of care received. In Canada, the Cultural Consultation Model provides cultural expertise, either by evaluating patients (preferably accompanied by the referring doctor, a translator and cultural mediator) for 1-3 sessions, providing recommendations to the referring doctor; or providing consulting to a referring doctor or organisation, through general guidance or discussion of specific cases.

**Conclusions:**

In an evergrowing globalization process, we will inevitably have more contact with patients from culturally distinct backgrounds. To provide the best care, we must be aware of the ways in which culture can shape symptom expression, and take into account cultural explanations and preferences.

**Disclosure:**

No significant relationships.

